# Association between cannabis use and major depressive disorder: a population-based cross-sectional study

**DOI:** 10.1186/s42238-026-00396-x

**Published:** 2026-04-14

**Authors:** Yanzhe Ning, Jigao Sun, Jiayan Zhou, Yihao Wang, Zucheng Shang, Mengfan Li, Hongzheng Li, Ya Xuan Sun, Ying Wu, Themistocles L. Assimes, Hui Zhang, Guang Chen

**Affiliations:** 1 Beijing Key Laboratory of Mental Disorders, Beijing Anding Hospital, National Clinical Research Center for Mental Disorders & National Center for Mental Disorders, Capital Medical University, Beijing, 100088 China; 2 Department of Orthopedics, Dongfang Hospital Beijing University of Chinese Medicine, Beijing, 100061 China; 3 School of Medicine, Stanford University, Stanford, CA 94305 USA; 4 Department of Psychiatry, The Second Affiliated Hospital of Naval Medical, University of the Chinese People’s Liberation Army, Shanghai, 200003 China; 5 Guang’anmen Hospital, China Academy of Chinese Medical Sciences, Beijing, 100053 China; 6 Fujian University of Traditional Chinese Medicine, Fujian, 350108 China; 7 T.H. Chan School of Public Health, Harvard University, Boston, 02115 USA; 8 Harvard Law School, Harvard University, Cambridge, 02138 USA; 9 Affiliated Hospital of Shaanxi University of Chinese Medicine, Xian, 712000 China; 10 Li Ka Shing Faculty of Medicine, The University of Hong Kong, Hong Kong, 999077 China

**Keywords:** Cannabis use, Major depressive disorder, Depression, Cross-sectional study, UK biobank

## Abstract

**Background:**

Major depressive disorder (MDD) is a chronic, recurrent psychiatric illness with challenging management, partly due to the heterogeneity of clinical presentations, unpredictable course and episodes, and variable treatment responses. Cannabis may alleviate depression by promoting glutamate, GABA, and dopamine, but results from previous studies remain controversial or inconsistent. In this study, we aimed to investigate the association between cannabis use and the prevalence and severity of MDD in adults.

**Methods:**

This study utilized data from the UK Biobank, with cannabis use as the exposure variable and depression as the outcome. Depression was ascertained using six distinct definitions, including broad clinical diagnosis, symptom-based criteria, self-reported depression, electronic health record-based diagnoses, and a strictly defined diagnostic category based on guideline criteria. C-reactive protein (CRP) levels and markers of biological aging were extracted from the database and analyzed as potential mediators. Both logistic and linear regression models were employed to examine the correlation. Subgroup analyses were used to explore potential effect modifiers while sensitivity analyses were used to ensure the robustness of the regression models.

**Results:**

A total of 33,749 individuals were included in the final analysis, with 50.5% being male. Cannabis use was associated with a 27% increase in the odds of depression prevalence (adjusted odds ratio [OR] = 1.27; 95% confidence interval [CI] = 1.18–1.38). No significant association was observed between the frequency of cannabis use and the number of depression episodes. Subgroup analyses revealed that, among current smokers, cannabis use was not significantly associated with increased odds of depression (OR = 1.09; 95% CI = 0.89–1.35). CRP levels may mediate this relationship, whereas markers of biological aging did not. Sensitivity analyses conducted a priori demonstrated consistent results, supporting the robustness of these findings.

**Conclusion:**

Cannabis use is associated with elevated odds of depression, with a comparatively attenuated impact observed among current smokers. However, the quantity of cannabis consumed did not correlate with the number of depression episodes. Further research employing longitudinal cohort studies or controlled clinical trials is needed to further inform evidence-based depression management strategies.

## Introduction

Major depressive disorder (MDD) is a chronic, recurrent psychiatric illness that significantly impairs psychosocial functioning and substantially diminishes quality of life (Malhi & Mann, 2018). As of 2008, MDD was ranked as the third leading cause of global disease burden and is projected to ascend to the top position by 2030 (Kessler and Bromet 2013), with a notable increase in incidence among young populations (Thapar et al. 2022). Approximately 20% of individuals will experience at least one episode of depression during their lifetime. Despite the availability of diverse treatment modalities—including psychotherapy (notably cognitive-behavioral therapy), pharmacotherapy (such as selective serotonin reuptake inhibitors), and neuromodulation techniques (e.g., transcranial magnetic stimulation)—the management of MDD remains challenging. This difficulty is partly attributable to the heterogeneity of clinical presentations, unpredictable course and episodes, and variable treatment responses (Marwaha et al. 2023). Notably, approximately 27% of patients fail to achieve remission following standard interventions, and the recurrence rate remains high, with nearly 80% experiencing subsequent episodes. Consequently, the therapeutic paradigm has shifted from merely eliciting a symptomatic response to attaining full remission, restoring functional capacity, and fostering resilience (Qaseem et al. 2023). Future strategies for MDD include prevention, early intervention, and comprehensive management, which are crucial for addressing its complex challenges.

MDD is a multifactorial condition resulting from an intricate interplay of genetic, environmental, psychological, and biological factors, which cannot be fully elucidated by any single hypothesis or theoretical framework (Marx et al. 2023). The predominant theories and hypotheses underlying the pathogenesis of MDD include the monoamine hypothesis, alterations in the hypothalamic-pituitary-adrenal (HPA) axis, neuro-inflammation, neuroplasticity deficits, and systemic influences (Cui et al. 2024). Although most currently effective antidepressants have been developed based on the monoamine hypothesis—such as adrenergic receptor antagonists and serotonin receptor agonists—this neurotransmitter-centric explanation does not account for the delayed onset of therapeutic effects, which often takes several weeks (Berton et al. 2006). This complexity may contribute to the observed low remission rates and high relapse rates following standard first-line treatments, underscoring the need for a more comprehensive understanding of MDD’s underlying mechanisms.

Cannabis, commonly known as marijuana, is derived from the Cannabis sativa plant, which has been cultivated for centuries for its psychoactive and medicinal properties (Ebbert et al. 2018). The principal psychoactive compound in cannabis is delta-9-tetrahydrocannabinol (THC), although the plant also contains other cannabinoids such as cannabidiol (CBD), which exert diverse physiological effects (Connor et al. 2021). Cannabis has been utilized for various purposes, including recreational use, pain management, and the alleviation of symptoms associated with chronic conditions such as epilepsy and multiple sclerosis (Baker et al. 2003). From a neurochemical perspective, the monoamine hypothesis of depression posits that imbalances in neurotransmitters like serotonin, norepinephrine, and dopamine are central to the pathogenesis of MDD. Cannabis compounds, notably THC and CBD, interact with the brain’s endocannabinoid system, which modulates neurotransmitter activity and may, therefore, influence these monoaminergic pathways. This interaction suggests a potential role for cannabis in the prevention and management of MDD. However, evidence regarding this relationship remains inconclusive. A meta-analysis of 11 longitudinal studies indicated that adolescent cannabis users (aged 18 years or younger) were approximately 1.4 times more likely to develop depression in young adulthood (ages 18–32) (Lydiard et al. 2023). Nonetheless, this association may be confounded by various factors, such as exposure to school bullying, family conflict, or minority sexual orientation—all of which are significant stressors linked to adult depression. Currently, the relationship between cannabis use and the risk or severity of depression remains unclear and requires further investigation.

Despite the absence of evidence supporting the use of cannabis for depression management, cannabis has become more prevalent in the United States following legalization in multiple states (Han and Palamar 2020). Additionally, studies have documented a significant rise in the social acceptability of recreational cannabis use in Canada (Doggett et al. 2025). However, many healthcare providers, including general physicians, report a lack of confidence in counseling patients regarding cannabis use and its potential mental health implications (Yusupov et al. 2025). This underscores the necessity for clear evidence and clinical guidance on cannabis consumption. Consequently, our study aimed to investigate the association between cannabis use and the prevalence and severity of MDD in adults, and to explore the underpinning biological and psychological mechanisms.

## Methods

### Participants

**Fig. 1:**
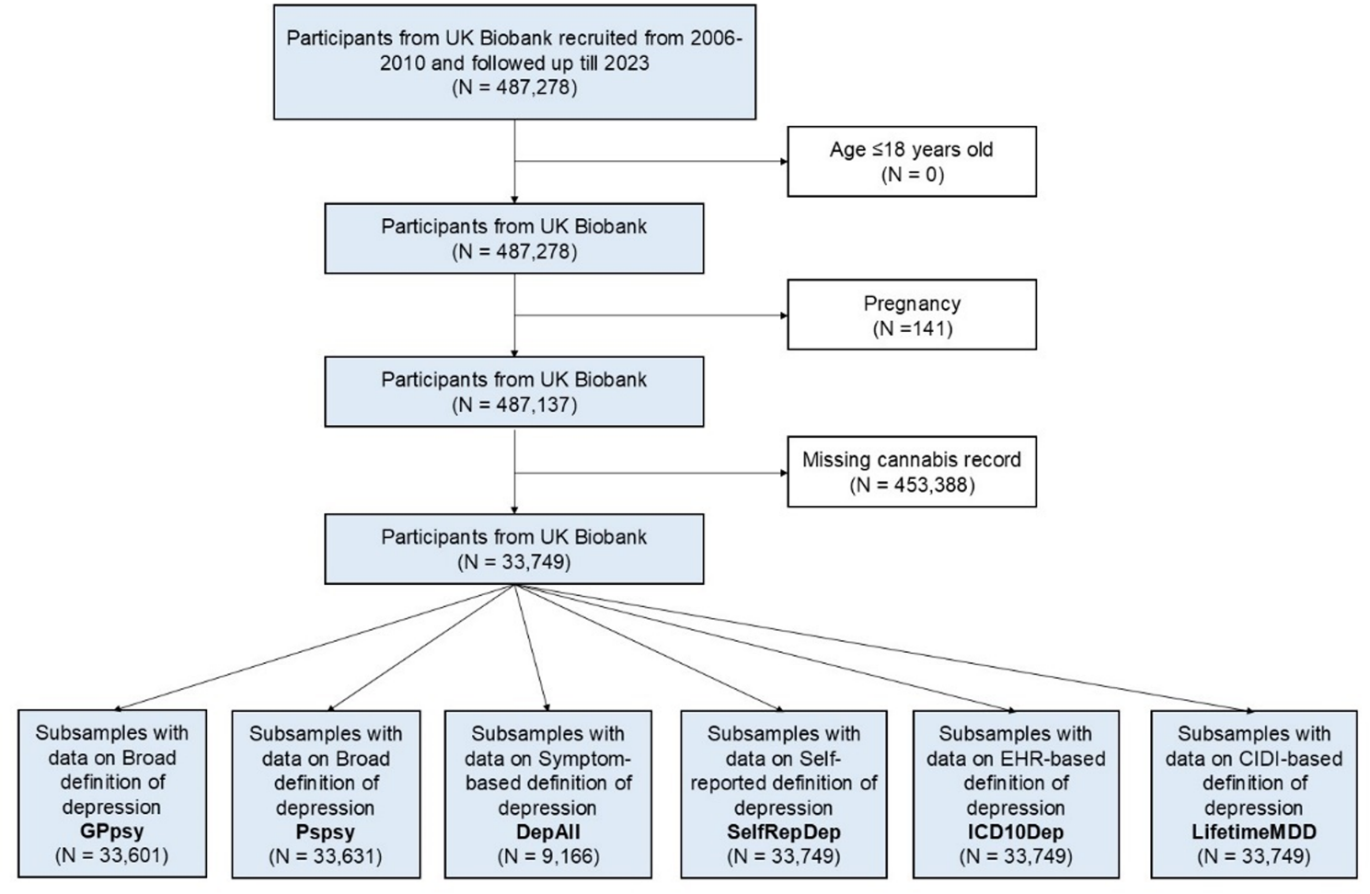
Flow chart for participant selection in UK Biobank

This study utilized data from the UK Biobank, a large-scale database that encompasses detailed demographic, environmental, and genetic information from participants. We conducted this cross-sectional analysis in accordance with the Strengthening the Reporting of Observational Studies in Epidemiology (STROBE) guidelines (S1 Checklist). From the UK Biobank database, we initially identified 487,278 UK adults who participated in the study since 2006. We excluded participants under the age of 18 and those who were pregnant, as well as individuals with missing data on cannabis use. After these exclusions, a total of 33,749 individuals were included in the analysis (Fig. 1).

### Exposure measurement

Cannabis use was identified as the exposure variable, extracted from Data Fields 20,453 and 29,104 in the UK Biobank database. Both fields recorded cannabis use as a binary variable (YES or NO). The data encompassed various forms of cannabis (e.g., marijuana, hash, skunk). Additionally, the frequency of cannabis consumption was obtained from Data Field 29,107 “Frequency of most regular cannabis use (which may be now)”, which was categorized into four groups: “Less than once a month,” “Once a month or more, but not every week,” “Once a week or more, but not every day,” and “Every day.”

### Outcome ascertainment

The outcome of this study was lifetime or broad definition of MDD, ascertained using six different definitions, based on methodologies outlined in previous study (Cai et al. 2020). These definitions are as follows: (1) Broad clinical definitions. GPpsy: Participants who reported seeing a general doctor for nerves, anxiety, tension, or depression, identified from Data Fields 2090. Psysy: Participants who reported seeing a psychiatrist for nerves, anxiety, tension, or depression, identified from Data Fields 2100. (2) Symptom-based depression (DepAll): Derived from responses to Data Fields 20,126, 4631, 5375, 4598, 4609, 2090, and 2100, capturing various depression-related symptoms and diagnoses. (3) Self-reported depression (SelfRepDep): Based on Data Field 20,002, where an illness code of 1286 indicates a self-reported diagnosis of depression. (4) Electronic health record (EHR)-based diagnosis (ICD10Dep): Identified via primary and secondary ICD-10 codes for depression (F32, F33, F34, F38, F39) in Data Fields 41,202 and 41,204. (5) Strictly defined lifetime MDD (LifetimeMDD): Based on the Composite International Diagnostic Interview Short Form (CIDI-SF), derived from Data Fields 20,440 and 20,442. The severity of depression was assessed based on the number of depression episodes, as recorded in Data Field 20,442, a continuous variable described as mean and standard deviation. By employing these multiple definitions, the study aims to comprehensively capture various aspects and diagnoses of depression within the UK Biobank cohort.

### Covariate extraction

Factors at baseline that were not equally distributed across cannabis groups and were considered to affect depression, were considered as confounding factors and were used as covariates in regression models, as outlined by the Directed Acyclic Graph (DAG) approach. In our study, confounding factors include sex (male/female), ethnic group, body mass index (BMI) (≤ 25, > 25 to < 30, ≥30 kg/m2), family income (average income), physical exercise (measured by Metabolic Equivalent Task), sleep disorder, smoking status, alcohol intake, and history of cancer diagnosis. Additionally, polygenic risk scores for bipolar disorder were extracted from the UK Biobank database and included as a covariate in the regression model to further mitigate potential bias, since no polygenic risk score for depression is available.

### Mediator calculation

Two potential mediators were identified and quantified. C-reactive protein (CRP) levels were directly obtained from the UK Biobank database. Biological aging was estimated using a composite biomarker derived from the following parameters: albumin, creatinine, glucose, lymphocyte percentage, mean cell volume, red cell distribution width, alkaline phosphatase, white blood cell count, and chronological age, as described in prior studies (Liu et al. 2018, Qiu et al. 2023). Any variable collected at baseline along with the exposure, such as smoking and sleep disturbance, was not considered a potential mediator because proper temporal ordering from exposure to mediator is required for mediation analysis.

### Statistical analysis

Baseline characteristics of participants with and without cannabis use were summarized using the *tableone* package in Python. Continuous variables were reported as means with standard deviations (SD) or medians with interquartile ranges (IQR), depending on their distribution. Categorical variables were presented as absolute counts with corresponding percentages. To assess differences between the two groups at baseline, independent *Student’s t-tests* were applied for continuous variables, and *Chi-squared tests* were used for categorical variables.

To investigate the association between cannabis use and depression, we employed three statistical models: a logistic regression model with cannabis use as an independent variable and depression diagnosis as dependent variable, a generalized linear regression model treating cannabis use as a binary independent variable and number of depression episode as dependent variable, and a generalized linear regression model treating frequency of cannabis use as an independent variable and number of depression episode as dependent variable. All analyses were adjusted for pre-specified measured confounding factors by adding covariates to the models. These models were adjusted for confounding factors such as sex, ethnicity, income, BMI, sleep disorder, smoking, alcohol intake, and history of cancer. We addressed missing data in covariates using multiple imputation prior to conducting the adjusted regression analyses. Multiple imputation was performed with the “mice” package in R, using “Predictive Mean Matching” for continuous variables, “Logistic regression” for binary variables, and “Polytomous logistic regression” for categorical variables. The imputed covariates included BMI, ethnic group, average total household income before tax, smoking status, alcohol intake, and physical exercise.

To identify potential effect modifiers in the relationship between cannabis use and depression, subgroup analyses were conducted by stratifying the analyses by sex (female vs. male), BMI (≤ 25, > 25 to < 30, ≥30 kg/m2), income level (average family income), alcohol status, smoking status, exercise status, and history of cancer diagnosis. To investigate potential mediators in the relationship between cannabis use and depression, mediation analyses were conducted using CRP and biological aging as mediator variables and taking age, sex, and BMI as confounding factors. To ensure the robustness of our findings, we conducted several sensitivity analyses by using different depression definitions as outcome variables in the regression models. This approach allowed us to assess the consistency of the associations across various operationalizations of major depressive disorder.

The statistical analyses were conducted in Python (Version 4.3.2), and Stata (Version 16.0). We used the Python package *tableone* package in Python for baseline characteristic analysis, the *glm* in R for generalized regression models, and the *mediation* in R for mediation analyses. All plots were created using R. Differences were considered statistically significant at a two-sided significance level of 0.05.

## Results

### Baseline characteristics

Table 1 summarizes the baseline characteristics of the participants included in this study from the UK Biobank according to their cannabis use status. A total of 33,749 individuals were included in the final analysis, with a mean age of 53.7 ± 7.5 years for participants without cannabis use and 51.8 ± 7.1 years for participants with cannabis use. The average BMI was 26.7 ± 4.6 kg/m2 for participants without cannabis use and 26.3 ± 4.4 kg/m2 for participants with cannabis use. Most of the included participants were British, comprising 88.8% of those without cannabis use and 85.4% of those with cannabis use. More than half of the participants with cannabis use were male (53.7%), while 53.9% of the participants without cannabis use were female. Among the included participants, 6.6% of those without cannabis use and 5.6% of those with cannabis use were cancer patients; 3.5% of those without cannabis use and 7.1% of those with cannabis use were current smokers; and 8.0% of those without cannabis use and 8.7% of those with cannabis use had trouble sleeping every day.

**Table 1:** Baseline characteristics of the included participants in UK biobank

Characteristics	Overall (*n* = 33,749)	Without cannabis use (*n* = 14,460)	With cannabis use (*n* = 19289)	*P* value
Age, mean ± SD, years	52.6 (7.3)	53.7 (7.5)	51.8 (7.1)	< 0.001
Sex, n (%)				
Male	17,030 (50.5)	6665 (46.1)	10,365 (53.7)	< 0.001
Ethnic group, n (%)				< 0.001
White	45 (0.1)	23 (0.2)	22 (0.1)	
Asian or Asian British	1 (0.0)	0 (0.0)	1 (0.0)	
Black or Black British	1 (0.0)	1 (0.0)	0 (0.0)	
Chinese	39 (0.1)	20 (0.1)	19 (0.1)	
British	29,294 (86.8)	12,842 (88.8)	16,452 (85.4)	
Irish	1085 (3.2)	412 (2.9)	673 (3.5)	
Any other white background	2199 (6.5)	773 (5.3)	1426 (7.4)	
White and Black Caribbean	80 (0.2)	23 (0.2)	57 (0.3)	
White and Black African	43 (0.1)	15 (0.1)	28 (0.1)	
White and Asian	113 (0.3)	45 (0.3)	68 (0.4)	
Indian	93 (0.3)	43 (0.3)	66 (0.3)	
Pakistani	18 (0.1)	5 (0.0)	13 (0.1)	
Caribbean	181 (0.5)	66 (0.5)	115 (0.6)	
African	59 (0.2)	22 (0.2)	37 (0.2)	
Other ethnic group	209 (0.6)	64 (0.4)	145 (0.8)	
Average household income (£), n (%)				< 0.001
Less than 18,000	3368 (10.0)	1351 (9.4)	2017 (10.5)	
18,000 to 30,999	5583 (16.6)	2481 (17.2)	3102 (16.1)	
31,000 to 51,999	8929 (26.5)	3856 (26.7)	5073 (26.4)	
52,000 to 100,000	10392 (30.9)	4344 (30.1)	6048 (31.5)	
Greater than 100,000	3762 (11.2)	1581 (11.0)	2181 (11.3)	
BMI, mean ± SD, kg/m2	26.5 (4.5)	26.7 (4.6)	26.3 (4.4)	< 0.001
MET minutes per week, mean ± SD	2326.3 (2,250.7)	2281.3 (2,216.9)	2359.5 (2,274.6)	0.003
Trouble in sleep, n (%)				0.072
Not at all	15802 (46.8)	6863 (47.5)	8939 (46.3)	
Several days	12467 (36.9)	5332 (36.9)	7135 (37.0)	
More than half the days	2598 (7.7)	1082 (7.5)	1516 (7.9)	
Nearly every day	2841 (8.4)	1163 (8.0)	1678 (8.7)	
Current smoking, n (%)				< 0.001
Never	28487 (84.5)	12896 (89.2)	15591 (80.9)	
Previous	3354 (9.9)	1042 (7.2)	2312 (12.0)	
Current	1877 (5.6)	512 (3.5)	1365 (7.1)	
Alcohol intake frequency, n (%)				< 0.001
Daily or almost daily	10001 (29.6)	4048 (28.0)	5953 (30.9)	
Three or four times a week	9820 (29.1)	4198 (29.0)	5622 (29.2)	
Once or twice a week	7537 (22.3)	3420 (23.7)	4117 (21.4)	
One to three times a month	3239 (9.6)	1465 (10.1)	1774 (9.2)	
Special occasions only	1989 (5.9)	885 (6.1)	1104 (5.7)	
Never	1126 (3.4)	436 (3.0)	699 (3.6)	
Cancer diagnosed by doctor, n (%)				< 0.001
No	31622 (93.7)	13463 (93.1)	18159 (94.2)	
Yes	2030 (6.0)	960 (6.6)	1070 (5.6)	

*BMI* Body Mass Index, *MET* Metabolic Equivalent Task

### Association between cannabis use and depression

Table 2 illustrates both the association between cannabis use and the prevalence of depression, as well as the correlation between the frequency of cannabis use and the number of depression episodes. Positive correlations were observed between cannabis use and depression prevalence; however, no significant correlation was identified between cannabis use frequency and the number of depression episodes. After adjusting for potential confounders—including sex, BMI, ethnicity, income level, smoking status, alcohol consumption, exercise habits, and cancer diagnosis—the regression analysis demonstrated that cannabis use was associated with a 27% increase in the odds of depression prevalence (adjusted odds ratio [OR] = 1.27; 95% confidence interval [CI] = 1.18–1.38).

**Table 2:** Association between cannabis use and depression

Depression outcome	Depression definition	Model I β (95% CI)	*P* value	Model II β (95% CI)	*P* value	Model III β (95% CI)	*P* value
Cannabis use on prevalence, OR	Lifetime MDD	1.23 (1.14, 1.32)	< 0.001	1.33 (1.23, 1.44)	< 0.001	1.27 (1.18, 1.38)	< 0.001
	GPpsy	1.18 (1.13, 1.24)	< 0.001	1.28 (1.22, 1.34)	< 0.001	1.25 (1.20, 1.31)	< 0.001
	Psypsy	1.36 (1.28, 1.45)	< 0.001	1.40 (1.31, 1.50)	< 0.001	1.37 (1.28, 1.46)	< 0.001
	DepAll	1.20 (1.05, 1.37)	0.009	1.29 (1.13, 1.48)	< 0.001	1.24 (1.08, 1.43)	0.002
	SelfRepDep	1.16 (1.00, 1.35)	0.051	1.19 (1.02, 1.38)	0.023	1.15 (0.99, 1.34)	0.074
	ICD10Dep	1.41 (0.88, 2.33)	0.165	1.49 (0.92, 2.47)	0.111	1.28 (0.78, 2.13)	0.339
Cannabis use on frequency, MD	Lifetime number of depressed episodes	0.13 (−0.09, 0.35)	0.256	0.13 (−0.10, 0.36)	0.257	0.12 (−0.11, 0.35)	0.299
Cannabis dose on frequency, MD	Lifetime number of depressed episodes	0.06 (−0.09, 0.22)	0.444	0.06 (−0.10, 0.22)	0.481	0.03 (−0.15, 0.22)	0.731

Model I: raw model without covariates to adjust; Model II: adjusted for gender, BMI, race, and income level

Model III: adjusted for covariates in model II and smoking, alcohol intake, physical exercise, history of cancer, and polygenic risk scores for bipolar disorder

*LifetimeMDD* Major depressive disorder diagnosed by CIDI criteria, *GPpsy* General doctor-defined depression, *Psypsy* Psychiatrist-defined depression, *DepAll* Depression defined by clinical symptoms, *SelfRepDep* Self-reported depression, *ICD10Dep* EMR-based depression

### Stratified subgroup analysis

**Fig. 2:**
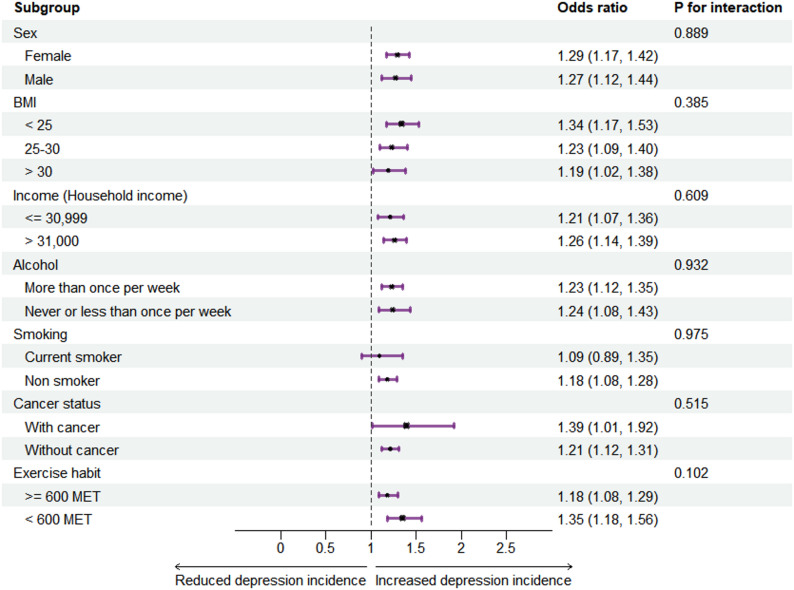
Subgroup analyses of the association between cannabis use and depression

Figure 2 examines potential effect modifiers in the relationship between cannabis use and depression. Subgroup analyses reveal that the association between cannabis intake and depression was consistent across various subgroups. Notably, among current smokers, cannabis use was not significantly associated with increased odds of depression prevalence (OR = 1.09; 95% CI = 0.89–1.35).

### Mediation analysis

**Fig. 3:**
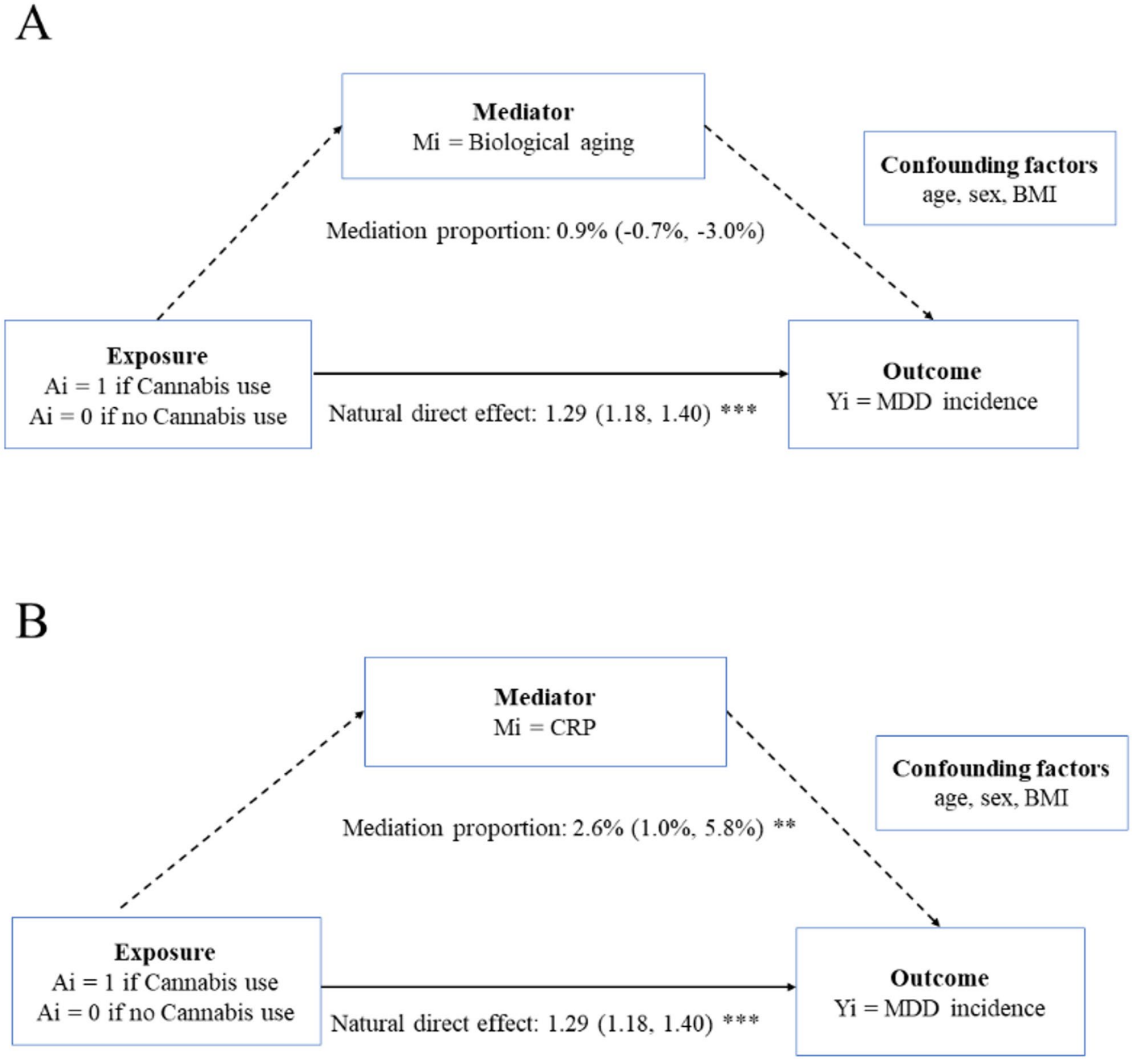
Mediation analysis of the association between cannabis use and depression. **A** Mediator of biological aging; **B** Mediator of CRP. *CRP* C-reactive protein, *MDD* Major depressive disorder, *BMI* Body mass index. *P < 0.05, **P < 0.01, ***P < 0.001

Figure 3 presents the results regarding exploratory mediation analyses of biological aging and the inflammatory biomarker CRP in the relationship between cannabis use and depression prevalence. A significant mediating effect of CRP was observed, with CRP accounting for approximately 2.6% of the association between cannabis use and depression prevalence (95% confidence interval [CI]: 1.0% to 5.8%; *P* < 0.01). In contrast, no statistically significant mediation effect was found for biological aging.

### Sensitivity analysis

The pre-specified sensitivity analyses yielded consistent results with the primary findings. When comparing depression defined by strict diagnostic criteria to alternative definitions—such as broad clinical categories (GPpsy and Psysy), symptom-based depression (DepAll), self-reported depression (SelfRepDep), and Electronic Health Record (EHR)-based diagnoses (ICD10Dep)—similar correlations with cannabis use were observed (Table 2).

## Discussion

### Summary of main findings

This study, utilizing large-scale cross-sectional data, found that cannabis use is associated with increased odds of depression; however, the quantity of cannabis used was not related to the number of depression episodes. Notably, although cannabis use is associated with a higher probability of depression prevalence, causal inference remains challenging. It is currently unclear whether cannabis use leads to an increase in depression or if pre-existing depression predisposes individuals to use cannabis.

### Comparison with earlier findings

With the progress of cannabis legalization, increasing numbers of individuals have access to cannabis (Hasin et al. 2025); however, whether cannabis has mood-enhancing effects or benefits for depression remains highly debated. Previous studies have demonstrated that: (1) depression is more likely to lead to cannabis initiation (Wang et al. 2025); (2) cannabis use significantly increases among individuals with depression (Myers et al. 2025); and (3) cannabis initiation can substantially reduce anxiety and depression symptoms (Wolinsky et al. 2025). Nonetheless, many studies indicate that the prevalence of depression is significantly higher among cannabis users (Churchill et al. 2025), and cannabis use is notably associated with suicidal behaviors (Maviel et al. 2025). Furthermore, long-term cannabis use has been linked to poor mental health (Matheson et al. 2025, Ricci et al. 2025). These inconsistent findings reflect the complexity of depression as a condition. In addition, it is important to distinguish more clearly between the acute effects of cannabis use and the consequences of long-term or habitual cannabis use. Our study clearly found that cannabis use is associated with an increased likelihood of depression, but higher quantities of cannabis consumption are not related to alleviation of depressive symptoms. This suggests that a higher frequency of cannabis use was not associated with fewer lifetime episodes, but the causal conclusion about treatment efficacy of cannabis use on depression cannot be drawn.

The interactions underlying the effects of cannabis and the molecular pathology of depression are highly intricate. The primary active constituents of cannabis are cannabinoids, such as THC and CBD. These compounds exert their effects mainly through interaction with endogenous cannabinoid receptor-1 and cannabinoid receptor-2 (CB1 and CB2) (Burggren et al. 2019). Previous research suggests that cannabinoids may alleviate depression by activating CB1 receptors, thereby inhibiting the release of various neurotransmitters—such as glutamate, GABA, and dopamine—and influencing neural signaling pathways that regulate pain, anxiety, and mood (Niesink & van 2013). Additionally, cannabinoids can impact synaptic plasticity and neuronal growth, potentially conferring neuroprotective effects (Van et al. (Van Vliet, et al. 2007)). CBD, in particular, may also exert anxiolytic and antidepressant effects by modulating non-cannabinoid receptors, including TRPV1 channels (Kaur et al. 2016).

However, our findings indicate that cannabis use does not correlate with the severity of depressive symptoms. This may be related to the currently unclear mechanisms underlying depression. The hypothesis that cannabinoids alleviate depression through activation of CB1 receptors, thereby suppressing neurotransmitter release, relies on the monoamine hypothesis of depression. Yet, this hypothesis has been widely challenged, as pharmacological agents developed based on it do not produce immediate clinical effects correlating with increased blood levels of monoamines; instead, therapeutic benefits typically manifest after several weeks of treatment (Berton & Nestler 2006). These findings are consistent with the broader recognition that monoamine-based models only partially explain depression.

Mediation results offer insights into both psychological and biological mechanisms that may underlie major depressive disorder and cannabis use. In addition to CB1, cannabinoids can also activate CB2 receptors, modulating immune cell function and reducing inflammation, which confers anti-inflammatory and antioxidant properties (Baron 2018). Our study observed that elevated CRP levels significantly mediated the association between cannabis use and depression, suggesting that inflammatory responses may play a role in the impact of cannabis on depression; however, no significant relationship was observed with markers of aging. Notably, these results may be confounded by variables such as smoking, alcohol consumption, and physical activity.

Furthermore, our data indicate that the correlation between cannabis use and increased risk of depression is weaker among current smokers. This suggests that the effects of cannabis may be similar to those of tobacco, potentially involving overlapping pathways that increase depression risk (Nguyen et al. 2023). When both exposures occur concurrently, there may be competitive or interactive effects influencing mental health outcomes.

### Limitations

Although this study employed a large cross-sectional study with an adequate sample size, controlled for multiple measured confounding variables, and demonstrated consistency through sensitivity analyses, several limitations should be acknowledged. First, the cross-sectional design restricts the findings to associations and precludes causal inferences. Second, recall bias may have influenced the results, as self-reported data on cannabis use from the UK Biobank questionnaires could be subject to inaccuracies or memory recall errors. Third, although a non-linear relationship between cannabis intake and depression is plausible, the lack of continuous variables—such as precise cannabis dosages or depression severity scores—limited the application of advanced modeling techniques, such as cubic spline analyses, to adequately explore potential non-linear associations. Fourth, the mediation analysis was considered exploratory because there was no clear temporal ordering between the exposure, mediator, and outcome variables. Fifth, this study only included UK Biobank participants, who are predominantly middle-aged, White, and healthier than the general population, which limits generalizability to younger, more diverse, or heavier cannabis-using populations.

### Future directions

Future research should consider the following directions: First, elucidate the causal relationship between routine cannabis use and the development of depression and other mental health disorders utilizing longitudinal data. Second, assess the potential therapeutic effects of cannabis initiation in reducing the frequency or severity of depression episodes. Third, investigate the underlying mechanisms by which cannabis influences depression, including testing various hypotheses related to depression pathology. These avenues are essential for advancing understanding and informing clinical use of cannabis products.

## Conclusion

In conclusion, cannabis use is associated with elevated odds of depression, with a comparatively attenuated impact observed among current smokers. However, the quantity of cannabis consumed did not correlate with the number of depression episodes. Further research employing longitudinal cohort studies or controlled clinical trials is essential to elucidate the causal relationships between cannabis use and depression, including whether depression predisposes individuals to initiate cannabis use and whether routine cannabis consumption alleviates depressive symptoms. Such investigations are critical for informing evidence-based management strategies tailored to populations vulnerable to depression.

## Data Availability

This research has been conducted using the UK Biobank Resource under Application Number 52374. Requests to access the data should be made via application directly to the UK Biobank https://www.ukbiobank.ac.uk/.
